# Treatment preferences in juvenile idiopathic arthritis – a comparative analysis in two health care systems

**DOI:** 10.1186/1546-0096-9-S1-P138

**Published:** 2011-09-14

**Authors:** Boris Hugle, Susanne M Benseler

**Affiliations:** 1 Division of Rheumatology, Hospital for Sick Children, Toronto, Canada; 2 Pediatric Rheumatology Hospital, Garmisch-Partenkirchen, Germany

## Background/aims

Variations in the treatment of Juvenile Idiopathic Arthritis (JIA) may impact on quality of care. The objective of this study was to identify and compare treatment approaches for JIA in two health care systems.

## Methods

Pediatric Rheumatologists in Canada (n=58) and Germany/Austria (n=172) were surveyed by email, using case-based vignettes for oligoarthritis and seronegative polyarthritis. Data was analyzed using descriptive statistics; responses were compared using univariate analysis.

## Results

Total response rate was 63%. Physicians were comparable by age, level of training and duration of practice, but more Canadians were based in academic centers. German physicians were more likely to institute DMARD treatment in oligoarthritis refractory to NSAID (p<0.001), and oral steroid treatment in uveitis (p=0.043). Canadian physicians were more likely to switch to a different DMARD rather than a biologic agent in polyarthritis refractory to DMARD. Both physician groups agreed on time to judge effectiveness of DMARDs (mean 4.2 months) and time to switch to biologic treatment (mean 5.4 months). 86% and 90% of German physicians preferred regular physiotherapy over home exercise compared to 14% and 15% in Canada for oligoarthritis and polyarthritis, respectively. Except for a Canadian preference for Naproxen in oligoarthritis, no significant differences were found for NSAID, intraarticular steroid preparations, initial DMARD and initial biologic treatment.

## Conclusion

Treatment of oligo- and polyarticular JIA with DMARD follows established guidelines, while usage of intraarticular steroids and biologic agents is variable within and between physician groups. Physiotherapy has a fundamentally different role in the two health care systems.

